# Managing clinical trials during COVID-19: experience from a clinical research facility

**DOI:** 10.1186/s13063-020-05004-8

**Published:** 2021-01-18

**Authors:** Frances Shiely, Jean Foley, Amy Stone, Emma Cobbe, Shaunagh Browne, Ellen Murphy, Maeve Kelsey, Joanne Walsh-Crowley, Joseph A. Eustace

**Affiliations:** 1 Trials Research and Methodologies Unit, HRB Clinical Research Facility at University College Cork, Mercy University Hospital, Grenville Place, Cork, Ireland; 2 School of Public Health, University College Cork, Western Road, 4th Floor Western Gateway Building, Cork, Ireland; 3 Department of Renal Medicine, Cork University Hospital, Cork, Ireland

**Keywords:** Trial management, Pandemic, Clinical trials, Participant safety, COVID-19

## Abstract

There is a dearth of literature on best practices for managing clinical trials, and little is understood on the role of the clinical trial manager. The COVID-19 pandemic has brought this into focus, and the continuance of clinical trials worldwide has been catapulted into a state of uncertainty as countries enter lockdown to manage the spread of the virus. Participant retention is an ongoing issue in clinical trials, and the concern is that in the current pandemic environment, attrition will be an issue which could potentially jeopardise trial completion. The current situation has necessitated timely problem solving by the trial manager to ensure trials remain open, and most importantly, that participant safety, paramount in clinical trials, is monitored. The purpose of our study is to highlight key issues arising in the management of clinical trials during a pandemic from first-hand experience in a clinical research facility managing both academic and commercial clinical trials. We offer some practical guidance on solution implementation.

## Background

Managing clinical trials efficiently is a necessary part of conducting clinical trials, not least because trials are run on a budget, but despite this we know very little about the practicalities of managing them competently. An editorial in *Trials* by Treweek and Littleford in 2018 highlighted the dearth of research on the management of clinical trials [1]. In the current climate of COVID-19, there has been a huge shift in how we manage clinical trials. Various challenges exist: government directed restriction on the movement of people; maintaining essential services only—is research an essential service?; risk of infection to trial participants and healthcare staff; requirement to self-isolate if ill; requirement to restrict movement if a close contact; and the knock-on effects of changes to staffing. Clinical trial managers are an important member of the trial team. They have a wealth of knowledge and expertise built up, and more attention needs to focus on sharing their expertise. While regulatory authorities in individual countries (the European Medicines Agency (EMA) [2], the Health Products Regulatory Authority (HPRA) (Ireland) [3], the Medicines and Healthcare Products Regulatory Agency (MHRA) (UK) [4], the Food and Drug Administration (FDA) (USA) [5]) have all responded with guidance to direct clinical trial managers during this pandemic, local solutions to some challenges are still necessary. The trial manager typically navigates between ethics committees, sponsors, PIs, hospitals and participants, to enable key decisions and/or actions in a timely and safe manner.

Literature has evolved, and continues to evolve, quickly during this pandemic, as globally the world unites to suppress transmission of the virus. Clinical trial managers, eager to share their experiences of dealing with COVID-19, have also made valuable contributions [6–9]. These papers vary from providing examples of changes to the management of specific trials, or managing a temporary trial suspension, to highlighting the importance of utilising new technologies and innovative methods to overcome challenges imposed by this pandemic in order to continue with data collection and other trial activities.

Our paper arises from experiences managing clinical trials in a clinical research facility in the South West of Ireland affiliated with three university hospitals. The clinical research facility employs approximately 40 people and manages both academic and commercial clinical trials, as well as observational studies. All academic trial recruitment was initially suspended, and only essential IMP (investigational medicinal product) trials continued. The current paper includes experiences of managing eight commercial clinical trials. These were all phase III multinational, IMP trials in dermatology (*n* = 1; total target recruitment (TTR) = 810), epilepsy (*n* = 2; TTR 555 and TTR 1000), peanut immunotherapy (*n* = 3; TTR = 350, TTR = 250 and TTR = 500), neurology (*n* = 1; TTR = 600) and cystic fibrosis (CF) (*n* = 1; TTR = 280). The purpose of our study is to add to the current literature on the day-to-day challenges experienced by trial sites, when managing clinical trials in a pandemic, to highlight some key issues facing clinical trial managers, and to discuss strategies that work when managing trials remotely, or with limited access. We provide a set of practical recommendations for clinical trialists managing clinical trials during a pandemic, based on our experiences of managing academic and commercial clinical trials in a clinical research facility during the COVID-19 pandemic.

## Trial management issues arising

### Leadership

A critical issue is to establish if research is an “essential service” at a time when the public health advice is to restrict the movement of people by enacting new laws to ensure they stay at home. Workers in this situation are also naturally worried about contracting COVID-19, given they are working in a hospital environment. Engagement with the clinical research facility and senior management at an early stage, and a joint vision that research is required (in the search for both treatments and vaccines) and is a necessary part of the solution to the crisis is critical. Encouraging, motivating and energising staff, as well as providing flexible working arrangements to allow them be part of, and contribute meaningfully, to delivering lifesaving treatments are essential. Flexibility from everybody in the organisation, management and workers, allows greater cover for trials over weekends/evenings and also helps with social distancing. It also ensures that if one member of staff gets COVID-19, it does not put the rest in isolation. Appropriating a schedule so that staff are not on site together is also important to decrease the risk of infection and also ensure there are back-up staff.

### Ethics

Ethical amendments were needed, and urgently. It was necessary for the sponsor to send amended documents into the ethics committee for review, e.g. letters to GPs, informed consent forms, protocols as well as a summary of guidelines for conducting trials during COVID-19. In the initial stages, an urgent safety amendment was submitted for the CF trial but changes were immediately effective. The dermatology trial required a protocol amendment. Amendments were also needed to get approval from the regulatory authority (HPRA) to get the IMP shipped to participants for the cystic fibrosis, immunotherapy and dermatology trials, as these participants were immunocompromised and were unable to come to the hospital for review. The CF participants fell into a category of participant for whom stopping their supply of IMP would have irreversible long-term consequences. We were strongly supported by our Ethics Committee which put in place an expedited review process and approval for contingency plans to keep trials running that had begun prior to the COVID-19 pandemic, as well as expedited review and approval for all COVID-19 studies. HPRA and ethics updates were forwarded by the trial manager to the study monitor for all eight trials, who in turn informed the sponsor of the updates. Though the ethics committee and the HPRA were notified that IMP would be shipped for participants, official approval of these notifications was not attained for 3/4 months after the initial notification, when the full new protocol amendment was approved. Directions from the regulatory authorities around the world on how to respond to the need for immediate change in trials during a pandemic is something that should be considered in the event that we experience a future pandemic.

### Data collection considerations

The main concern was how to input data when access to notes was not possible and how to capture a participant’s visit when it is a virtual visit? There have been modifications to data collection procedures which varied depending on trial type. The sponsor in the cystic fibrosis and epilepsy trials recommended telephone calls to collect some data, and questionnaires were posted to participants at home with the instruction to the participant to return them when they attend for the next hospital visit. For the dermatology trial, a telephone call was made to participants to review adverse events (AEs) and suspected adverse events (SAEs). In the epilepsy trials, participants sent pictures of their seizure diary via email as required, though this procedure was in place prior to the pandemic. Likewise, for the immunotherapy trials, participants routinely take and forward pictures if any AEs occur, so this did not change in lockdown. In one of the immunotherapy trials, the participants have daily electronic diaries to complete. These are monitored from site for compliance, AEs, tolerance of IP and concomitant medications at all times. Also in the immunotherapy trials, the doctor did an assessment and filled out a modified case report form (CRF) which was sent to the sponsor by email, who only approved the shipment of the IMP when the assessment was complete. Trialists need to consider a move to electronic CRFs, to avoid the challenges experienced due to data collection on paper, experienced during this pandemic.

The most important data collection during a pandemic is safety information, i.e. AE, SAE and any change in medication. However, not all sponsor guidelines instruct the participant to attend for safety bloods. To attend for bloods was a decision agreed between the PI and participant in the cystic fibrosis trial. In the dermatology trial, having safety bloods completed was a requirement prior to sending IMP while for the epilepsy studies, though decision-making regarding attendance for safety bloods was with the PI, there were no safety concerns because no new AEs were reported by the participant, so no participant returned on site. For the immunotherapy studies, safety bloods were postponed. There remains a risk benefit consideration regarding having a participant attend either the hospital or GP surgery for safety blood tests, which probably needs to be individualised for each trial and potentially each participant. This should be future proofed, such that in the event of a pandemic or other issue preventing participants from travelling, there is consideration of a contingency plan such as the possibility of home testing as appropriate.

### Training, communication and management of the trial team

Training for the trial team was moved to online via Skype, Zoom or MS Teams, whichever worked best in the hospital or clinical research facility setting. Correspondence between monitors, sponsors, labs and external companies moved to email for all eight trials. Early engagement with the trial sponsor was also crucial. Regular communication with the trial team was necessary to keep everyone engaged and up to date, but varied from trial to trial. The CF trial communication was a minimum of twice weekly, and the dermatology team communications were decided on by the trial coordinator. For the epilepsy studies, updates were provided to the team as often as updates came from the sponsor or the HPRA, and for the immunotherapy trials, the team were in contact daily. For all eight trials, the sponsors sent updates on trial conduct via email. New worksheets were created for visits which contained new orders for procedures. For all trials, a procedure for consent to share personal information with the sponsors was identified. A training call arranged by the sponsor took place before all procedures began. New training logs had to be signed by all the team. In this instance, motivation of the trial team was a concern initially, but it was not an issue in our clinical research facility which we believe is due to continued regular engagement with all staff, both managerial and operational, and the flexibility afforded. Staff recruitment did not arise, but should it arise during a pandemic, remote interview procedures would be necessary.

A study phone was carried by the trial team 24/7, and the responsibility was divided between the team. Management of this study phone was also remote—study phone was diverted to a personal phone.

In a pandemic situation, a communication strategy for managing trials in a crisis within the clinical research facility as well as individually for each trial is essential.

### Recruitment

Recruitment of new participants for all pre-existing clinical trials, both academic and commercial, ceased completely. This decision was made by the PI in all instances, and participant safety was cited as the reason. The HPRA in Ireland issued guidance that supported this “The ability to confirm eligibility, and to conduct key safety assessments and study evaluations, is of particular importance. Where required, recruitment should be temporarily halted, or suspended and subjects discontinued” [3]. This was common across the globe. In the UK, reducing face-to-face contact was prioritised to safeguard participant *and staff* safety [6]. In England, the NHIR reported that during the peak of the pandemic of 3906 non-commercial studies that were open or in set-up during the height of the pandemic, 70% of those had to be paused and only 12% remained open to recruitment during the peak of the outbreak [10]. Reduced and restricted recruitment caused by the pandemic has affected all IMP studies [11] and has been documented in a variety of fields [12–14], but the effects of the pandemic are not limited to these fields and have likely affected all areas of research. Existing studies of therapeutic interventions, where the PI assessed it is in the best interest of the subject to continue with the trial, continued but with some difficulties. All on-site monitoring visits, other than remote monitoring, were cancelled. The dermatology and epilepsy trials continued. In neurology, infusions continued but were delayed due to reduced capacity because of social distancing requirements. In cystic fibrosis, therapeutic interventions continued, but were conducted remotely at the participant’s residence. Any site visit had to be risk assessed for all parties regarding potential cross. In the absence of on-site visits for all eight trials, trial participants were followed up by phone with the research nurse and PI. Recruitment of participants for COVID-19 studies began and is ongoing.

After 6 weeks in the first lockdown, there was a shift in the response to the pandemic by the ethics committee and ethics applications for trials other than COVID-19 trials were modified to allow for telephone clinical visits where the trial would be introduced by the clinician during a standard of care visit. The research team would then follow up with potential participants by telephone to explain the trial in more detail. The PIL and consent were posted to participants and returned by mail. Questionnaires were completed by participants at home and returned by mail, and participant notes and lab results were reviewed in the hospital. Setting up studies, reviewing budgets, contracts, ethics etc. for new non-COVID-19 studies is also important so the clinical research facility is in a state of readiness when the restrictions are lifted.

### Retention

Over time, the limited ability to perform safety assessments is likely to affect retention so regular contact with participants is necessary to keep them safe and engaged. We have not had a retention issue to date (9 months since first lockdown), but it is monitored closely. All remote “visits” are entered in the CRF as protocol deviations. One particular neurology trial involved 6-monthly infusions. These have not been able to go ahead as the participants are immunocompromised and it was not safe for them to attend the hospital during the COVID crisis. The sponsor was in agreement and asked the PI to determine when it is safe for participants to return to the hospital. Six months after the first lockdown, the PI deemed it safe for the participants to return to hospital to receive their infusion and continue on in the trial. We were asked by the CF sponsor to explore the option of home visits for safety and endpoint assessments. This necessitated getting indemnity to cover staff to perform home visits.

In the initial stages of the pandemic, the commercial funders were anxious to continue with participant visits. A risk assessment was conducted by the PI for each of the trials and on-site visits continued until it was deemed no longer safe. All visits stopped during the week of 16 March 2020, which was when the government directed a lockdown for the entire country. Thereafter, participant information was collected electronically or over the phone. For the CF trial and dermatology trials, guidance on safety monitoring for participants in order to allow them to continue on the studies was provided by the sponsor. The epilepsy studies were withdrawn by the sponsor (due to analysis from the previous phase 2 trials), so participants were brought on site for close out visits.

Though retention is not an issue at our clinical research facility, literature tells us that existing restrictions and difficulties adhering to trial protocols will lead to missing data and trial drop-outs due to difficulties in conducting follow-up appointments. There is limited literature available on the extent of this effect to date, but the amount of missing data in trials is likely to be larger than normal due to the pandemic [15]. A survey assessing the impact of the pandemic on RCTs on acute ischemic stroke and cerebral aneurysms found that trial teams reported follow-ups have been impacted with many participants missing trial-related clinics and follow-up appointments [16]. Many clinical trial teams, however, have adopted new practices and adjusted their trial activities to facilitate the running of trials in the current climate to insure data collection from already enrolled trial participants by using new technologies, innovative methods and “telehealth” approaches [6, 9, 11, 12].

### Managing IMP

The sponsor outlined their contingency plan complying with national regulatory bodies, i.e. the HPRA in Ireland. If the participant was unable to attend the hospital for the visits—a family member could collect the IMP, or the site could ship IMP directly to the participant. The decision to ship IMP was decided early in the pandemic by the PI and sponsors based on benefit/risk considerations for the trial participants. It was challenging to manage in some instances, as not all pharmaceutical companies involved in the commercial trials are proactive. For the multinational dermatology trial, where safety bloods are analysed in another venue in Europe, there was a break in the chain of delivery, due to the closing of borders. To ensure participant safety, local labs were arranged to process the safety bloods.

A courier was arranged by the pharma company to get the IMP shipped to the participant at the correct temperature, i.e. ambient or refrigerated. Bookings for IMP shipment were arranged by site staff for all trials. In the case of the CF, epilepsy and dermatology trials, consent was verbal and followed up with written consent once amendment for these changes was approved. For the dermatology trial, the research nurse sought permission from the participant via email. In the case of the paediatric trials, consent was gained via email from the parents of the trial participants, for the purpose of sharing their contact details with the courier. Ethical and HPRA approval was sought and given prior to any of these procedures taking place. The participant was contacted by phone to see if the drug arrived intact. All of this was documented in the source notes. Participants were initially consented for storage of their personal data in the hospital. By introducing an external body, i.e. a courier company, their personal data was leaving the site which necessitated their consent to be documented in their medical notes.

### Study samples

The contingency plan for frozen samples from studies was that they would remain on site and would be forwarded when travel restrictions were lifted. Safety bloods continued as normal.

### Monitoring and audit

Monitors were not allowed on site. Monitoring visits were all done remotely. All required data will be source data verified during on-site monitoring visits post-COVID-19. Site staff were available to the monitor by email. The monitor was in regular contact with the trial manager for all trials. They asked for scans of updated training logs, informed consent trackers, delegation logs and even renewed GCP certificates when they expired. The clinical research facility introduced a new online GCP training course in response to these requests. In our experience, some monitors were asking for non-essential items and asking to file in the site file. Given the current unprecedented working arrangements, maintenance of a site file, in our view, is not an essential priority and all documents can be filed when the restrictions are lifted. Some commercial study companies are developing processes around these procedures. We anticipate this will change policy on risk and mitigation in the future.

### Recommendations

At the time of writing, Ireland has entered a second level 5 lockdown. The major change in procedures between lockdown one and lockdown two is to monitoring and patient visits. Monitors continue to come on site in this second lockdown. Anybody that comes on site to the clinical research facility completes the mandatory COVID-19 symptom questionnaire. Patient visits continue on site for those wishing to come in. Those that prefer not to come in, or are deemed at too high a risk by the PI, continue with remote visits. Table 1 presents the recommendations for managing clinical trials during a pandemic.

**Table 1:** Recommendations for managing clinical trials during a pandemic

**Leadership**	Priority setting within host institutions/clinical research facilities that research is an essential service and a necessary part of the solution to the public health crisis
	Mobilisation of the team and organisation of flexible work schedules for the full team to maintain study flow, as well as facilitate staff who have childcare issues due to the closing of childminding facilities. Rotations and back-up staff schedules are also necessary to allow for the fact staff may need to self-isolate. Ensuring all staff are not on site together to minimise the possibility of contraction of the virus is critical.
	Ongoing risk assessment due to the rapidly changing environment and public health advices.
	Encouragement, motivation and energising staff through regular communication and providing social support.
**Communication**	Communication strategy within the host institution/clinical research facilities to keep staff engaged.
	Establishment of virtual meetings by Skype/Zoom/MS Teams study training sessions, and for sharing information and mitigating risk.
	Daily conference calls should take place between the trial team, so the workload is distributed amongst all staff equally. Subject-specific issues can also be discussed during this time.
	All emails should be cc’d with all nurses to ensure no data is lost in the event of staff becoming unwell.
	Establishment of an online telephone group for the team, e.g. WhatsApp, which is critical for the social support of staff.
	Establishment of virtual meetings by Skype/Zoom/MS Teams study for virtual coffee/social support (non-work related activities).
	Maintaining contact with other research centres is important to troubleshoot as well as provide moral support in an evolving crisis.
	An extra level of collaboration is required between the medical staff and the trial manager to facilitate conducting remote visits and safety assessments prior to IMP distribution.
	Regular communication with trial monitors is essential.
**Ethics**	Establishment of an expedited ethics approval locally is paramount as a change in protocol is required in order to ensure studies can proceed.
	Email integrity is crucial at this time as there are a lot of emails going to participants and separately going to sponsors and this may inadvertently cause an error in sharing identifying information or sharing information with staff who should remain blinded.
**Technological supports**	Establishment of remote access for many systems, e.g. establishment of remote access to the hospital laboratory system and also ITU to monitor COVID-19 patients remotely.
	Access to a secure shared drive to store trial documents so they can be accessed remotely and securely as most work is done from home.
	Remote access for CRF Manager room booking so that staff can work in the hospital and keep a safe distance from each other.
	Online GCP programme is necessary.
	There may be difficulty printing emails due to restrictions of office space. A study folder should be created, and all documents stored there until access to a printer is available.
**Engagement with regulatory authorities**	Supplying ongoing IMP to participants is vital. This will need to be carefully considered to maintain the blinding of the sponsor, i.e. not providing names and addresses, to maintain research integrity, and to ensure safety of both staff and participant at all times.
	Receipt of IMP should be verified by staff via phone call to the participant, or the participant’s parents if a minor.
	Shipments should be clustered so as to limit staff time in the hospital and thus protect their safety. Deliveries should be limited to specified days also.
**Participant engagement**	Contact with participants to ensure retention is critical.
	A study phone should be carried by one member of the team to cover 24/7 contact from trial participants. This should be shared amongst the trial team, i.e. trial managers, research nurses, PIs and co-PIs.

## Conclusion

Managing clinical trials is a multifaceted job, and many challenges arise, even in “normal” circumstances. Managing clinical trials during a pandemic adds an additional layer of complexity. This study is based on the experiences of clinical trials staff who have adapted to managing academic and commercial trials during the COVID-19 pandemic, in a clinical research facility. Further research is necessary to document the workload of clinical trial managers, to fully understand their role and its critical interface with clinical trial participants, sponsors, regulatory authorities and other clinical trial staff. We recommend that in the future, when seeking ethical and regulatory approval, all studies should have a contingency plan for situations where participant movement may be restricted.

## Data Availability

Not applicable.
